# A cognitive versus behavioral approach to emotion regulation training for externalizing behavior problems in adolescence: Study protocol of a randomized controlled trial

**DOI:** 10.1186/s40359-018-0261-0

**Published:** 2018-10-10

**Authors:** L W te Brinke, H D Schuiringa, A T A Menting, M Deković, B O de Castro

**Affiliations:** 1 Department of Developmental Psychology, Utrecht University, Heidelberglaan 1, 3584 CS Utrecht, The Netherlands; 2 Department of Clinical Child and Family Studies, Utrecht University, Heidelberglaan 1, 3584 CS Utrecht, The Netherlands; 3 Utrecht University, PO BOX 80140, 3508 TC Utrecht, The Netherlands

**Keywords:** Externalizing behavior, Aggression, Emotion regulation, Cognitive behavior therapy, Intervention components, Adolescence

## Abstract

**Background:**

Interventions for adolescents with externalizing behavior problems are generally found to be only moderately effective, and treatment responsiveness is variable. Therefore, this study aims to increase intervention effectiveness by examining effective approaches to train emotion regulation, which is considered to be a crucial mechanism involved in the development of externalizing behavior problems. Specifically, we aim to disentangle a cognitive and behavioral approach to emotion regulation training.

**Methods:**

A randomized controlled parallel-group study with two arms will be used. Participants are adolescents between 12 and 16 years old, with elevated levels of externalizing behavior problems. Participants will be randomly assigned to either the control condition or the intervention condition. Participants in the intervention condition receive both a cognitive and behavioral emotion regulation module, but in different sequences. Primary outcome measures are emotion regulation skills, emotion regulation strategies, and externalizing behavior problems. Questionnaires will be completed at pre-test, in-between modules, and post-test. Moreover, intensive longitudinal data is collected, as adolescents will complete weekly and daily measures.

**Discussion:**

Gaining insight into which approaches to emotion regulation training are more effective, and for whom, is important because it may lead to the adaptation of effective intervention programs for adolescents with externalizing behavior problems. Eventually, this could lead to individually tailored evidence-based interventions.

**Trial registration:**

The trial is registered at the Central Committee on Research Involving Human Subjects (NL61104.041.17, September 20th, 2017) and the Dutch Trial Register (NTR7334, July 10th, 2018).

## Background

If left untreated, externalizing behavior problems are a serious risk factor for the development of adverse outcomes later in life, such as rejection by peers, school failure, crime involvement and psychopathology [1–3]. Costs to society are estimated to be 10 times higher for youth with elevated levels of externalizing behavior problems than for typically developing youth [4]. Over the past years, knowledge regarding the effectiveness of interventions for externalizing behavior problems in adolescence has increased. These interventions are, however, still found to be only moderately effective and treatment responsiveness is variable [5, 6]. Therefore, this study aims to increase intervention effectiveness by examining effective approaches to train a crucial mechanism involved in behavior problems: emotion regulation.

Emotion regulation is a multidimensional construct, that is defined as the extrinsic and intrinsic processes responsible for monitoring, evaluating, and modifying emotional reactions [7]. Emotion regulation skills entail both the overall trait-level difficulties in regulating emotions (emotion regulation difficulties) and the habitual use of specific adaptive or maladaptive emotion regulation strategies (e.g., rumination) [8]. Both aspects of emotion regulation are found to be related to the development of externalizing behavior problems [9]. For example, emotion regulation difficulties predict increases in aggressive behavior during adolescence [10, 11], whereas the use of adaptive emotion regulation strategies (such as problem solving) are related to less psychopathology [12, 13]. The interplay between the use of adaptive (e.g., problem solving) and maladaptive (e.g., rumination) emotion regulation strategies is also important. Specifically, research shows that for adults who report to use high levels of maladaptive strategies, the use of adaptive strategies is negatively related to problem behavior, whereas this association is non-significant for participants who report to use low levels of maladaptive strategies [14]. So, the use of adaptive emotion strategies might have compensational effects. Similar results are found in adolescents. For example, adolescents who report to use a maladaptive emotion regulation profile (high use of maladaptive emotion regulation strategies combined with the low use of adaptive strategies) are specifically at risk for experiencing externalizing behavior problems [15].

Given the association between emotion regulation and externalizing behavior problems, it is not surprising that aspects of emotion regulation training (e.g., anger management, cognitive problem solving) are incorporated in many evidence-based interventions that aim to decrease externalizing behavior problems [16, 17]. For example, of all interventions targeting externalizing behavior problems in adolescence that are described in recent literature, 75% include an emotion regulation component [16]. In addition, research shows that incorporating aspects of emotion training increases treatment effectiveness [18]. A meta-analysis that investigated the effectiveness of Cognitive Behavioral Treatment (CBT) for anger in children and adolescents showed that the broadly defined construct ‘skills training’ (that includes emotion regulation skills training) was significantly more effective than affective education [18]. It is important to note, however, that these meta-analyses look at broadly defined common components, which, in addition to emotion regulation training, also include for example social skills training or exposure. Moreover, the approaches to train emotion regulation differ. Therefore, we do not know whether different approaches to emotion regulation training are equally effective for all adolescents.

An important differentiation among training approaches seems to be a focus on cognitive emotion regulation (e.g., cognitive reappraisal or problem solving) or behavioral emotion regulation (e.g., behavioral distraction or skills training) [19]. Evidence from literature on coping shows that cognitive and behavioral aspects can be disentangled [20] and that behavioral coping training might be more effective for adolescents than cognitive coping training [21]. However, coping refers to processes that are generated in response to stressful events, whereas emotion regulation refers to responses that are specifically aimed at the response to and modulation of emotions [22]. Results from the coping literature might therefore not be generalizable to the construct emotion regulation. Moreover, adolescents with externalizing behavior problems may have characteristics that make them more or less susceptible to specific training approaches. To our knowledge, the differences in effects between cognitive and behavioral emotion regulation training have not yet been investigated for adolescents with externalizing behavior problems.

On the one hand, indirect evidence suggests that behavioral emotion regulation training might be more effective than cognitive emotion regulation training. Sukhodolsky and colleagues [18] argued that CBT components that were “more behavioral” (e.g., skills development) seemed to be more effective than components that were “less behavioral” (e.g., problem solving). This implicates that treatments that teach actual behaviors might be more effective than treatments that attempt to modify internal constructs. This may pertain particularly to adolescents with behavior problems, who may be less susceptible to cognitive approaches than others because they are on average more impulsive, less verbally intelligent, and less self-critical than their peers [23]. On the other hand, there is also evidence that behavioral training is less effective than cognitive training for adolescents with externalizing behavior problems. Specifically, a meta-analysis by Candelaria and colleagues [24] found that anger management interventions for children and adolescents that used role play (a behavioral technique) were relatively ineffective, compared to other methods such as teaching problem solving or emotional awareness. It has been argued that specific behavior training transfers less to other situations than changing fundamental underlying cognitions. Another possibility is that behavioral and cognitive training approaches are only effective when they are combined, because they supplement or reinforce each other. This is in line with the notion that CBT is developed to integrate cognitive and behavioral therapeutic approaches [25]. If both approaches are useful, the sequence in which they are used may also influence effectiveness. It might be that cognitive changes only transfer to changes in behavior when cognitive training is followed by behavioral exercises. Alternatively, it might be that abstract cognitive instructions are only properly understood after behavioral exercises have made participants familiar with emotion regulation.

The current study therefore aims to examine which approach (cognitive or behavioral emotion regulation training) is more effective in improving emotion regulation skills and reducing externalizing behavior. To this end, we designed an experimental emotion regulation training (the Think Cool Act Cool training) consisting of two modules: cognitive training and behavioral training. These modules are presented to participants in different sequences to examine which (combination of) approaches improve emotion regulation skills and decrease externalizing behavior problems. With this experimental design we aim to test the direct effects on emotion regulation and externalizing behavior problems in order to examine relative contributions. The experiment is not intended to have the pervasive long-lasting effects of comprehensive multi-component interventions and does therefore not include follow-up assessments. To examine changes in emotion regulation and externalizing behavior problems, we will use baseline to post-intervention assessments, and intensive longitudinal data. Specifically, participants will report on weekly changes in aggression and emotion regulation. This allows us to examine dynamic within-subject changes in response to specific training experiences. In addition, this study incorporates a daily diary assessment, in order to examine whether emotion regulation training also effects mood variability. This is important, because emotional dynamics such as mood variability are viewed as an aspect of emotion regulation [26] and research shows that higher mood variability is associated with increases in externalizing behavior problems [26, 27].

In addition, this study will look at the effects of emotion regulation training on comorbid internalizing problems. Research shows that externalizing behavior problems frequently co-occur with internalizing problems such as anxiety and depression [28–30]. A factor that might underlie this co-occurrence is emotion regulation. Emotion regulation is proposed to be a transdiagnostic factor, that relates to heterotypic continuity across externalizing and internalizing behavior problems [8]. For example, a longitudinal study showed that for early adolescent boys, the emotion regulation strategy rumination mediated the transition from aggressive behavior to anxiety symptoms [30]. Given the transdiagnostic nature of emotion regulation, it is possible that an emotion regulation training that aims to decrease externalizing behavior problems, also effects comorbid internalizing problems. If this is the case, a transdiagnostic emotion regulation treatment approach might result in greater treatment efficacy for comorbid conditions [31].

In summary, emotion regulation training is a core component for the treatment of externalizing behavior problems in adolescence, but it is unclear whether cognitive and/or behavioral approaches make this component effective. Therefore, we aim to disentangle the effects of cognitive and behavioral emotion regulation training with an intensive longitudinal experiment. Important moderators and mediators will be taken into account to asses why and for whom which approach is effective.

### Hypotheses

We hypothesize that the Think Cool Act Cool emotion regulation training is effective in improving emotion regulation skills and decreasing externalizing behavior problems, compared to care-as-usual. We also hypothesize that the training has a small effect on mood variability and comorbid internalizing problems. In addition, we compare the contrasting hypotheses that the cognitive (Think Cool) module is more effective than the behavioral (Act Cool) module or vice versa and hypothesize that completing both modules is more effective than completing only one module. In addition, we compare the contrasting hypotheses that it is more effective to first receive the cognitive module and secondly the behavioral module (sequence Think Cool + Act Cool) or vice versa (sequence Act Cool + Think Cool). We expect that overall, emotion regulation mediates the effect of the Think Cool Act Cool training on externalizing behavior problems. In particular, we expect that behavioral emotion regulation mediates the effect of the Act Cool module on externalizing behavior problems and that both cognitive emotion regulation and social information processing mediate the effects of the Think Cool module. Regarding moderation effects, we expect that overall, the Think Cool Act Cool training is more effective for adolescents who report higher levels of affective reactivity, and for adolescents whose parents show more acceptance and less rejection [32, 33]. In addition, we expect that the Think Cool module is more effective for adolescents with higher intelligence, whereas the Act Cool module is more effective for adolescents with lower intelligence [23, 34]. Finally, we expect that higher treatment integrity is related to increased effectiveness [35].

## Method/design

### Study design

**Fig. 1:**
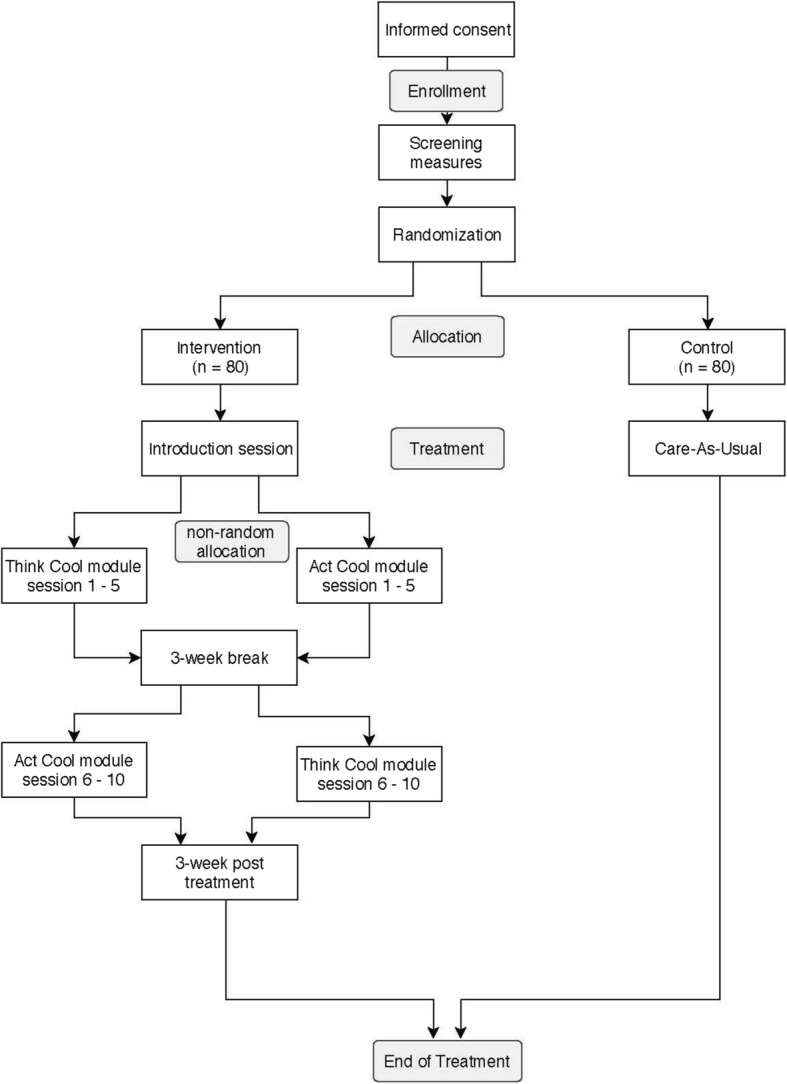
Overview of study design

This study is a randomized controlled parallel-group experiment with two conditions and two arms in the intervention condition. Participants are randomly assigned to either the control condition or the intervention condition. Participants in the intervention condition receive both the cognitive and behavioral module, but in different sequences. Specifically, participants in the intervention condition follow either first the cognitive and then the behavioral module (first treatment arm) or the reverse sequence (second treatment arm). In order to minimize contamination between the cognitive and behavioral module, individual participants in the intervention condition are not randomly assigned to a training sequence. Participants in the intervention condition from the same location (i.e. school) who start with the training at the same time (i.e. wave) follow the same sequence. In successive waves at the same school, the sequence will be reversed. An overview of the study design is presented in Fig. 1. Ethical approval for this study was granted by an independent medical ethics committee of the University Medical Center Utrecht.

### Eligibility criteria

Participants are recruited from Dutch high schools. Participants are between 12 and 16 years old, with elevated levels of externalizing behavior problems. The following inclusion criteria will be used: a subclinical or clinical level of externalizing behavior problems as reported by teachers (TRF externalizing subscale >84th percentile) and average or above average intelligence (estimated IQ score > 80). Participants are excluded if they experience severe Autism Spectrum symptoms as reported by their teacher (ASV symptom score > 98th percentile) and/or if their language, auditory or visual skills are severely hindered (as evidenced by an indication of the school psychologist that the adolescent possesses insufficient Dutch language skills to understand questionnaires and training, or has an auditory or visual disability). Participants with mild Autism Spectrum symptoms (ASV symptom score < 98th percentile) and/or other comorbid psychiatric problems (e.g., depression, ADHD) are not excluded from participation in this study.

### Sample size

The sample size of this study is based on the expected difference on the primary outcome variables (emotion regulation and externalizing behavior problems) between the intervention condition (both sequences together) and the control condition. Meta-analyses demonstrated that the expected effect size (*d*) of cognitive behavioral therapy for children and adolescents with externalizing behavior problems is between 0.25 and 0.30 [5, 6]. To detect a small to medium effect (Cohen’s *d* = 0.25–0.30), with a two-sided type I error rate of 0.05, a power of 0.95, and three measurement moments, we will need between 100 and 142 participants [36]. To account for dropout, we have determined the total sample size to be 160 (80 participants in the control condition and 80 participants in the intervention condition).

Because previous research did not investigate differences between cognitive and behavioral training modules, it is not possible to estimate the expected effect size for the difference between modules. However, a sensitivity-power analyses showed that with 80 participants in the two intervention arms, an error rate of 0.05, a power of 0.95, and 19 repeated weekly measurements, even small effect sizes of 0.09 can be demonstrated with within-subjects analyses [36].

### Procedure and randomization

**Fig. 2:**
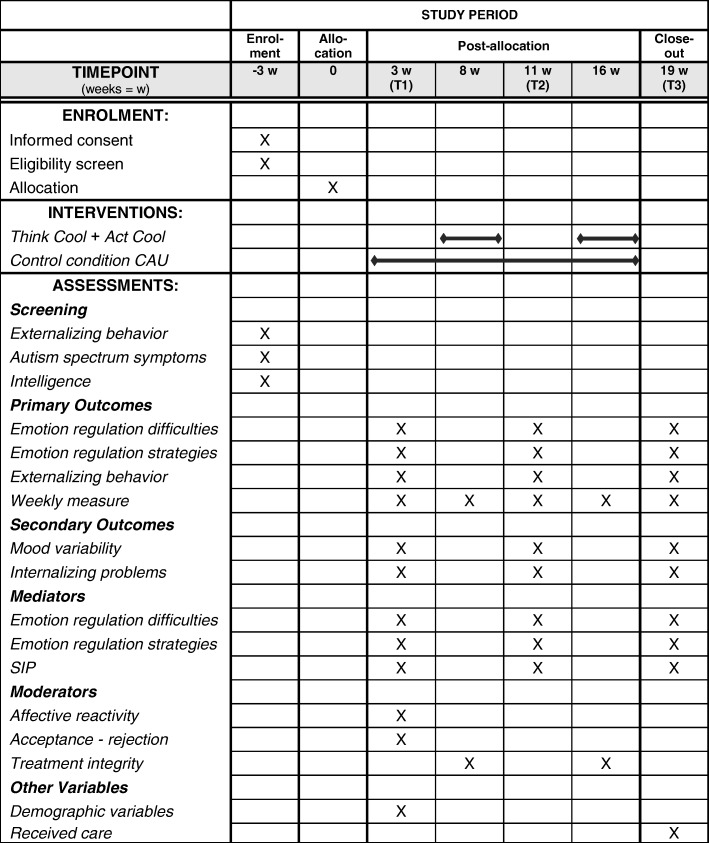
Spirit diagram. *Note. CAU* Care as Usual

First, participating schools send an information letter and consent form to all possibly eligible adolescents and their parents. After informed consent is obtained from both the adolescent and the parent(s) of adolescents aged 12–15 (for adolescents aged 16 informed consent of a parent was not required), teachers fill out the screening measures (externalizing behavior problems and severity of autism spectrum symptoms, see screening measures). Next, information about the adolescent’s intelligence is provided by the school. If information about IQ is not available or is derived from an intelligence test administered more than 2 years ago, a short IQ test will be administered. Fig. 2 shows the trial process with a Standard Protocol Items Recommendations for Interventional Trials (SPIRIT) figure.

If participants meet the inclusion criteria, they are randomly assigned to either the intervention or the control condition. Randomization takes place at the individual level, by means of computer-generated random numbers. Adolescents, their parents and teachers will obviously notice the condition in which they are participating, so allocation will not be blind. Nevertheless, participants will not be aware of the fact that we examine the difference between two training sequences. Subsequently, adolescent download a questionnaire application on their smartphone and start with the weekly and daily questionnaires. First, a 3-week baseline of the weekly measure (see measures section) will be established. Moreover, adolescents fill in the first Daily Diary measure on five consecutive days. In addition, adolescents, their parents and teachers complete the baseline measures at T1, the first of three assessments. The adolescent questionnaires and tasks are administered individually at school by a trained research assistant at each assessment point. Adolescents fill out the questionnaires on a computer. Teachers fill out the questionnaires on paper. Parents are sent links to the questionnaires via email.

Participants in the intervention condition start with either the cognitive module (Think Cool) or the behavioral module (Act Cool). After 5 weeks, in which participants in the intervention condition follow five individual therapy sessions, all participants, parents and teachers complete the T2 measures. Next, there is a 3-week training break, which allows us to measure possible delayed effects. During the training break, all participants continue to fill in the weekly questionnaire and fill in the second Daily Diary measure. Subsequently, participants in the intervention condition follow the second module (Think Cool or Act Cool, depending on the first module), which also consists of five individual sessions. Eventually, the post-test measures are completed by all participants at T3. There also is a 3-week post-measure of the weekly measure, in which participants also complete the third Daily Diary measure.

### Experimental and control condition

#### Experimental manipulation

Participants in the intervention condition will receive 11 individual 45-min sessions of the Think Cool Act Cool emotion regulation training. This is a manualized experimental training, that is designed based on components of evidence-based treatments for adolescents with externalizing behavior problems, such as Coping Power [37] and Aggression Replacement Training [38]. The training is provided at the school of the participant, by a trained clinician with a background in child psychology.

Before the actual modules, participants start with an introduction session, in which they get to know the trainer, the content of the training, and set personal goals. Next, participants first receive either the Think Cool module or the Act Cool module, followed by the other module. Both modules consist of five individual sessions. The content of the modules is displayed in Table 1.

**Table 1:** Content of the Think Cool Act Cool emotion regulation training

Session	Session components Think Cool module	Session components Act Cool module
Introduction session	• participant and clinician get to know each other • training objectives are explained • brainstorm about words for anger • formulate personal training goals	
Session 1 / 6	• make or adjust^a^ an anger thermometer, based on situations, bodily sensations and *cognitions* • explain the *Think* Cool Chain • practice with regulation strategy ‘*think* about something fun’ (cognitive distraction) • introduce at-home assignments	• make or adjust^a^ an anger thermometer, based on situations, bodily sensations and *behaviors* • explain the *Act* Cool Chain • practice with regulation strategy ‘*do* something fun’ (behavioral distraction) • introduce at-home assignments
Session 2 / 7	• look back and discuss at-home assignments • practice regulation strategy *‘talk in your head’* (cognitive relaxation) • practice regulation strategy *‘helping thoughts’* (cognitive reappraisal) • summarize and discuss new at-home assignment	• look back and discuss at-home assignments • practice regulation strategy *deep breathing* (behavioral relaxation) • practice regulation strategy *‘time out’* (behavioral modification) • summarize and discuss new at-home assignment
Session 3 / 8	• look back and discuss at-home assignment • practice to look at a situation from multiple viewpoints • introduce *cognitive* problem solving • practice perspective taking • summarize and discuss new at-home assignment	• look back and discuss at-home assignment • practice *behavioral* problem solving skills (set a boundary, ask for help, ask for an explanation) • summarize and discuss new at-home assignment
Session 4 / 9	• look back and discuss at-home assignment • practice cognitive problem solving • summarize and discuss new at-home assignment	• look back and discuss at-home assignment • practice behavioral problem solving in difficult situations (accusations, disappointments, frustration) • summarize and discuss new at-home assignment
Session 5 / 10	• look back and discuss at-home assignment • practice complete *Think* Cool Chain	• look back and discuss at-home assignments • practice complete *Act* Cool Chain

*Note:* ^a^ During the first session of the second module, the existing thermometer is adjusted. Therefore column “cognitions / behaviors” from the thermometer that was developed in the first session of the first module, is removed and a new column is added. Besides this, the sessions are the same, irrespective of the sequence in which the modules are followed

In both modules, adolescents are instructed to make daily at-home assignments, the “anger thermometer logbook”, in which they briefly describe in which situations they became angry and what strategies they used to regulate their anger and solve the issues. The situations they describe in the logbook are used in the training sessions as practice material. If adolescents do not complete the at-home assignment, clinicians use other situations from adolescents’ lives.

##### Think cool

In this module, participants learn cognitive emotion regulation strategies. The module is based on the Think Cool Chain, and consists of a cognitive approach to emotion regulation that is typically used in current interventions (e.g., [39, 40]). The first step of the chain (session 1) is to signal anger, with an anger thermometer that is based on situations, feelings, sensations and cognitions (e.g., “they always blame me”). Adolescents also learn to identify the “tipping” point, the point on the thermometer where it is smart to use one of the emotion regulation strategies. The second step of the chain is to practice three cognitive emotion regulation strategies (cognitive distraction, cognitive relaxation and cognitive reappraisal). Adolescents practice with these strategies in session 1 and 2. The third step of the chain is cognitive problem solving, which is practiced stepwise in session 3, 4, and 5. Adolescents learn specific cognitive problem-solving skills (understand a problem from multiple perspectives, think about possible solutions and possible consequences of these solutions, decide which is the most suitable solution) and practice these skills in a stepwise manner with paper-and-pencil exercises.

##### Act cool

In this module, participants learn behavioral emotion regulation strategies with the Act Cool Chain, consisting of a behavioral approach to emotion regulation that is typically used in current interventions (e.g., [40, 41]). The first step (session 1), is to signal anger with an anger thermometer, similar to the thermometer that is used in the Think Cool module. However, in the Act Cool module the thermometer is based on behaviors (e.g., “if I become angry I raise my voice”) rather than cognitions. The second step of the chain is to practice behavioral emotion regulation strategies (behavioral distraction, behavioral relaxation and time out). Adolescents practice these strategies in session 1 and 2. The third step of the chain is behavioral problem solving, which is practiced with behavioral exercises in session 3, 4, and 5. Adolescents learn specific behavioral skills (set a boundary, ask for help, ask for an explanation) and practice with difficult situations (accusations, disappointments, frustration).

##### Clinician training and supervision

Clinicians providing the experimental training receive a two-day training course, guided by the developers of the training manual. The training course starts with an introduction providing information regarding the theoretical background of the modules and practical tips with regard to the implementation of the modules. On the first training day, the focus is on the Think Cool module whereas the second training day focuses on the Act Cool module. In the afternoon session, clinicians practice their training skills by participating in and reflecting on role-plays. Moreover, the training course focuses on differentiation between cognitive and behavioral approaches, creating a safe atmosphere, motivating adolescents, explaining exercises, and discussing at-home assignments. During the intervention period, clinicians participate in at least two 3-h supervision sessions in which clinicians bring in topics that they would like to discuss or practice, and reflect on their skills. In addition, clinicians are able to receive consultation by phone on request.

#### Control condition

Participants in the control condition will receive care-as-usual (CAU). CAU is defined as the standard care that is available at school for all adolescents with behavior problems. This includes, for example, behavior management techniques provided by teachers (e.g., reinforcing positive behavior). Moreover, participants in both conditions are not withheld to receive other kind of help, if necessary (e.g., psychopharmaca). The received CAU and additional help will be measured and reported.

### Measures

All constructs, measures and informants are summarized in Table 2.

**Table 2:** Overview of measures and informants

Variable	Concept	Measure	Informant
Eligibility screening	Externalizing behavior	TRF	Teacher
	Autism Spectrum Problems	ASV	Teacher
	Intelligence	WISC-III-NL	Adolescent
Primary outcomes	Emotion regulation difficulties	DERS	Adolescent
	Emotion regulation strategies	FEEL-KJ, Vignette	Adolescent
	Externalizing behavior	YSR, TRF, CBCL	Adolescent, teacher, parent
	Weekly primary outcomes	Weekly questionnaire	Adolescent
Secondary outcomes	Mood variability	Daily Diary	Adolescent
	Internalizing problems	YSR	Adolescent
Mediators	Emotion regulation difficulties	DERS	Adolescent
	Emotion regulation strategies	FEEL-KJ, Vignette	Adolescent
	Social information processing	SIVT	Adolescent
Moderators	Affective reactivity	ARI-S	Adolescent
	Parental acceptance-rejection	PARQ	Parent
	Intelligence	WISC-III-NL	Adolescent
	Treatment integrity	TIQ, audiotapes	Clinician
Other variables	Demographics	Questions	Adolescent, parent
	Received care	Questions	School psychologist

*Note. TRF* Teacher Report Form, *ASV* Autisme Spectrum Vragenlijst, *YSR* Youth Self Report, *CBCL* Child Behavior Checklist, *DERS* Difficulties in Emotion Regulation Scale, *FEEL-KJ* Fragensbogen zur Erhebung der Emotionsregulation bei Kinder und Jugendlichen, *SIVT* Sociale Informatie Verwerkings Test, *ARI-S* Affective Reactivity Index, *PARQ* Parental Acceptance-Rejection Questionnaire, *TIQ* Treatment Integrity Questions

#### Screening measures

##### Externalizing behavior problems

Teachers will report on the externalizing behavior problems of the adolescent with the externalizing subscale of the Teacher Report Form age 6–18 [42]. This scale consists of 32 items (e.g., “Fights a lot”) that are rated on a 3-point scale from 0 *(not true)* to 2 *(very true or often true).*

##### Severity of autism spectrum symptoms

The severity of autism spectrum symptoms will be measured with the teacher reported *Autisme Spectrum Vragenlijst* [43]. This questionnaire consists of 24 items (e.g., “Exhibits odd, repetitive behaviors”) on a 5-point scale from 1 *(totally not agree)* to 5 *(totally agree).*

##### Intelligence

Intelligence will be assessed with the Dutch version of the Wechsler Intelligence Scale for Children (WISC-III-NL) [44, 45]. If the WISC-III-NL was completed by the adolescent within 24 months before the start of the study, this total IQ score will be used. If this score is not available, the subtests “Block Design” and “Vocabulary” will be completed by the adolescent. Subsequently, global intelligence will be estimated, based on the sum of the scaled subtest scores, with the formula for approximation of Full Scale IQ (FIQ) [46]. FIQ estimates are found to be reliable and strongly correlated with the total IQ score [47, 48].

#### Primary outcome measures

##### Emotion regulation difficulties

The Dutch version of the brief Difficulties in Emotion Regulation Scale (DERS) will be used to measure emotion regulation problems [49, 50]. The DERS is a 15-item self-report measure that assesses difficulties in emotion regulation. The items (e.g., “When I am upset, I become out of control”) are rated on a 5-point scale from 1 *(almost never)* to 5 *(almost always).*

##### Emotion regulation strategies

Emotion regulation strategies in response to feelings of anger will be assessed with the Dutch version of the Fragensbogen zur Erhebung der Emotionsregulation bei Kinder und Jugendlichen (FEEL-KJ) [51]. The subscale anger is assessed in this study and consists of 30 items (e.g., “If I feel angry… I do something fun”) that are rated on a 5-point scale from 1 *(never)* to 5 *(almost always).* The questionnaire distinguishes adaptive and maladaptive emotion regulation strategies.

In addition, cognitive and behavioral emotion regulation strategies will be measured with a newly developed vignette measure. The measure is based on earlier vignette measures [12, 52]*.* The adolescent reads a vignette that is meant to elicit feelings of anger, and rates how likely it is that he/she will use a specific emotion regulation strategy, on a 7-point scale from 0 *(definitely not)* to 6 *(definitely).* Per vignette, there are six behavioral strategies (adaptive strategies: relaxation, behavioral distraction, social support; maladaptive strategies: direct expression, indirect expression, avoidance), and six cognitive strategies (adaptive strategies: cognitive reappraisal, cognitive distraction, putting into perspective; maladaptive strategies: self-blame, rumination, suppression).

##### Externalizing behavior

Externalizing behavior will be measured from a multi-informant perspective, with subscales of the ASEBA-questionnaires that are administered to adolescents, their teachers, and parents [42]. Adolescents (YSR), Teachers (TRF), and Parents (CBCL) will complete respectively the 32, 32, and 35 items of the externalizing scale of the Dutch ASEBA versions [53]. Items (e.g., “Fights a lot / I fight a lot”) are rated on a 3-point scale from 0 *(not true)* to 2 *(very true or often true).*

##### Weekly measure

Emotion regulation and aggression will also be assessed with a 6-item self-reported weekly measure. The questionnaire contains three items for emotion regulation (e.g., “how often this week did you become *so* angry, that you could not control yourself?”) and 3 items for aggression (e.g., “How often did you hit someone this week?”) that are rated on a 5-point scale from 0 *(never)* to 4 *(more often, … times).* The measure is based on items of the DERS and YSR [42, 49].

#### Secondary outcome measures

##### Mood variability

Mood variability will be measured with the Daily Mood Device, an adapted version of the Electronic Mood Device [54, 55]. In the current study, the mood variability measure is integrated in the weekly measure smartphone application. At each measurement moment, adolescents are asked to rate the intensity of their daily mood for happiness, sadness, anger, and anxiety (“Today I feel …”) on five consecutive days. Each mood state will be measured with three items (12 items in total), that are rated on 9-point scale from 1 *(not happy / angry / …*) to 9 *(happy / angry / …)*. The words that are used for happiness are “glad”, “happy”, and “cheerful”, for sadness: “sad”, “down”, and “dreary”, for anger: “angry”, “cross”, and “short-tempered”, and for anxiety: “afraid”, “anxious”, and “worried”.

##### Internalizing problems

Internalizing problems will be reported by the adolescents with the internalizing scale of the Youth Self Report age 11–18 [42]. This subscale consists of 34 items (e.g., “I cry a lot”) that are rated on a 3-point scale from 0 *(not true)* to 2 *(very true or often true).*

#### Potential mediators

Emotion regulation skills (see for measures the primary outcome section) and social information processes are viewed as protentional mediators for models in which the effects of the Think Cool Act Cool training on externalizing behavior problems are tested.

##### Social information processing

Social information processing skills biases and deficits will be assessed with the *Sociale Informatie Verwerkings Test* (SIVT) [56]. The SIVT consists of six videos that show hostile, ambiguous or accidental interpersonal problems, involving a peer or adult perpetrator. In all videos, the outcome of the situation is negative for the victim. Different steps of social information processing (encoding, interpretation, goal setting, response generation, response evaluation and selection) are measured with a semi-structured interview and multiple-choice questions. In the current study, only ambiguous and accidental situations will be used because earlier research shows that with hostile situations, aggressive and non-aggressive are not very well distinguishable [57]. At each time point, the adolescent will view two videos; an ambiguous and an accidental situation with both a peer and adult perpetrator, but the order will be counterbalanced.

#### Potential moderators

##### Affective reactivity

Reactivity will be assessed with the Affective Reactivity Index (ARI-S) [58]. The ARI-S is a 6-item self-report measure that assesses irritability (e.g., “I often lose my temper”) on a 3-point scale from 0 *(not true)* to 2 *(certainly true).*

##### Parental acceptance-rejection

Parental acceptance-rejection will be measured with 18-items of the short version Parental Acceptance-Rejection Questionnaire (PARQ) [59]. Parents will report on three subscale of the PARQ; warmth, neglect and undifferentiated rejection (e.g., “I say nice things about my child”). Items are rated on a 4-point scale from 1 *(almost never true)* to 4 *(almost always true).*

##### Treatment integrity

Treatment integrity is conceptualized in this study as the extent to which the intervention is implemented as intended [60]. To measure treatment integrity, clinicians will fill in a questionnaire after each session. The questionnaire is based on other measures of treatment integrity [60–62] and consists of several domains; treatment exposure, treatment adherence, and treatment differentiation (e.g., “It was difficult to focus on behavior rather than cognitions in this session”). The questionnaire also measures participant comprehension and responsiveness (e.g., “The adolescent participated actively in this session”). In total, the measure consists of approximately 25 items, depending on the content of the session. Items are answered on 4-point scale from 1 *(not at all)* to 4 *(totally).* Moreover, all training sessions will be audiotaped. A random selection of 10% of the sessions will be scored on different aspects of treatment integrity (e.g., adherence, differentiation) by independent coders.

#### Other information

Demographic information (gender, ethnicity and socioeconomic status) will be assessed at baseline. In addition, the received care-as-usual and additional help will be measured at T3.

### Analyses

Data will be analyzed according to the intention-to-treat principle [63], with multiple imputation as technique to handle missing data. To answer the first research question, whether the Think Cool Act Cool emotion regulation training is effective in enhancing emotion regulation skills and decreasing externalizing behavior problems, data of T1-T3 will be analyzed with analysis of variance and/or structural equation modeling. We will examine whether different aspects of emotion regulation and multi-informant perspectives of externalizing behaviors problems can be combined into latent variables. If this is the case, these latent variables will be used, in structural equation models. Otherwise, the analyses of variance will be conducted separately for the different constructs. To examine which module (Think Cool versus Act Cool) and which sequence most effectively increases emotion regulation capacities, we will use piecewise growth curve analyses and analysis of variance. Moderation will be tested by using multi-group analyses or regression analyses, and mediation will be tested with random-intercept cross-lagged panel models and parallel-process piecewise latent growth curve modeling. The analyses and reporting of results will be carried out according to the Consolidated Standards of Reporting Trials (CONSORT) [64].

## Discussion

The goal of the current randomized controlled parallel-group study is to examine the effects of the Think Cool Act Cool emotion regulation training. Zooming in on the component emotion regulation allows us to make inferences about the efficacy of this specific treatment component. This will supplement the literature, because current knowledge about intervention component efficacy is mainly based on meta-analyses and reviews, and although these studies inform us which components are associated with larger program effectiveness, they do not allow to make causal inferences [65]. Moreover, the present study examines the differential effects of cognitive and behavioral emotion regulation training. As current interventions for adolescents with externalizing behavior problems are generally found to be only moderately effective [5], this knowledge is important, because it can lead to the future adaptation of current intervention programs.

A specific strength of the current study is that it includes the use of intensive longitudinal data, which allows us to examine dynamic within-subject changes. An additional advantage of this assessment method is that the weekly and daily diary questionnaires are less retrospective than regular measures and therefore might be less susceptible to recall bias [66]. Moreover, the current study will use multiple sources of information, as externalizing behavior problems will be reported by adolescents, parents, and teachers.

Despite the strengths and innovative aspects of the current study, there are some issues that the study is not able to take into account. Because the study does not include a condition in which adolescents receive only the behavioral or the cognitive module, we will not be able to examine follow-up effects of the separate training modules. Nevertheless, as the goal of the current study is to examine direct effects, we also do not intend to examine long-lasting effects. Another limitation of the study is the open design, as adolescents and other informants included in the assessments (parents and teachers) are aware of the fact that they are either in the control or intervention condition. Nevertheless, adolescents are not aware that we examine the difference between two training sequences.

In conclusion, the intensive longitudinal experiment that is described in this protocol will provide valuable information for both research and clinical practice, as it may inform the adaptation of intervention programs for adolescents with externalizing behavior problems. Gaining insight into which emotion regulation training approaches are more effective, and for whom, will eventually enable us to develop more effective individually tailored interventions.

## Abbreviations

ARI-S: Affective reactivity index
ASV: Autisme spectrum vragenlijst
CBCL: Child behavior checklist
CBT: Cognitive behavioral treatment
CONSORT: Consolidated standards of reporting trials
DERS: Difficulties in emotion regulation scale
FEEL-KJ: Fragensbogen zur Erhebung der Emotionsregulation bei Kinder und Jugendlichen
FIQ: Full scale IQ
PARQ: Parental acceptance-rejection questionnaire
SIVT: Sociale informatie verwerkings test
SPIRIT: Standard protocol items recommendations for interventional trials
TIQ: Treatment integrity questions
TRF: Teacher report form
WISC: Wechsler intelligence scale for children
YSR: Youth self report
